# A cross-sectional study on the association of serum uric acid levels with depressive and anxiety symptoms in people with epilepsy

**DOI:** 10.1186/s12888-020-03019-8

**Published:** 2021-01-07

**Authors:** Rui Zhong, Qingling Chen, Mengmeng Li, Nan Li, Chaojia Chu, Jing Li, Xinyue Zhang, Weihong Lin

**Affiliations:** 1 Department of Neurology, The First Hospital of Jilin University, Chang Chun, China; 2 Department of Hepatology, Tianjin Medical University, Tianjin Second People’s Hospital, Tianjin, China

**Keywords:** Serum uric acid, Epilepsy, Depressive symptoms, Anxiety symptoms, Serum

## Abstract

**Background:**

High serum uric acid (SUA) levels may provide protection against depression and anxiety through its defensive role in oxidative damage. The aim of this study was to test the hypothesis of the independent associations of lower SUA levels with depressive and anxiety symptoms among patients with epilepsy (PWE).

**Methods:**

A cross-sectional study was performed among 320 PWE aged ≥18 years old in Northeast China. The Neurological Disorders Depression Inventory for Epilepsy (NDDI-E; Chinese version) and the Generalized Anxiety Disorder-7 scale (GAD-7; Chinese version) were used as screening tools for depressive and anxiety symptoms for PWE. Serum uric acid levels were measured. The associations of SUA levels with depressive and anxiety symptoms were assessed by using binary logistic regression models, with adjustment for the related risk factors (*P*< 0.05).

**Results:**

Lower SUA tertiles were significantly associated with higher C-NDDI-E and GAD-7 scores compared with the higher two tertiles (*p*=0.001, and *p*= 0.002). Patients with depressive symptoms exhibited significantly lower SUA levels compared to those without depressive symptoms (*p*< 0.001). SUA levels of patients with anxiety symptoms were significantly lower than those of patients without anxiety symptoms (*p*< 0.001). The first and second SUA tertiles were associated with depressive symptoms, with the third tertile group as the reference group, after adjusting for confounders (first tertile: OR = 4.694, 95% CI = 1.643~ 13.413, *P* = 0.004; second tertile: OR = 3.440, 95% CI = 1.278~9.256, *P* = 0.014). However, The first and second SUA tertiles were not associated with the risk of anxiety symptoms compared with the third tertile in the adjusted logistic regression model (First tertile: OR = 1.556, 95% CI = 0.699~3.464, *P* = 0.279; second tertile: OR = 1.265, 95% CI = 0.607~2.635, *P* = 0.530).

**Conclusion:**

We found that lower SUA levels were independently associated with depressive symptoms but not with anxiety symptoms among PWE. Further well-designed prospective cohort studies are required to determine the causality of the associations and to further clarify the mechanisms of SUA in depressive symptoms.

## Background

Epilepsy is one of the most common severe brain diseases and is characterized by a lasting predisposition to generating spontaneous epileptic seizures [1]. Epilepsy has been reported to affect more than 70 million individuals worldwide, constituting a major contributor to the global burden of neuropsychiatric disorders [1, 2]. Patients with epilepsy (PWE) have an increased prevalence of psychiatric comorbidities [3]. Depression and anxiety are the most common psychiatric comorbidities among PWE [3–5]. Approximately one third of PWE were diagnosed with depression or anxiety during their lifetime [6]. Even the mechanisms underlying the relationship between depression and epilepsy have not been properly investigated. Increasing evidences have indicated that depression and epilepsy such as temporal lobe epilepsy (TLE) represent an epiphenomenon sharing similar neural networks [7]. Mental health was related to poorer quality of life and worse prognosis in people with epilepsy [8–10]. These psychiatric comorbidities have a huge burden on patients, families and society [11]. Considering the high prevalence and significant burden of affective disorders in PWE, it is of importance to find more risk factors for psychiatric comorbidities in order to improve preventive strategies and find new therapeutic targets.

Serum uric acid (SUA), the end-product of purine metabolism, has been known as an important natural antioxidant in the human body [12]. Recent progress has indicated that oxidative stress may be involved in the pathogenesis of depression and anxiety [13, 14]. Lowered SUA levels have been reported as a potential contributor to the development of depression and anxiety [15, 16]. A large scale study based on 2875 subjects concluded that individuals with lower plasma uric acid levels have a higher risk of current but not remitted major depressive disorders and/or anxiety disorders, and this finding showed the benefit of uric acid on mental health [16]. In a cohort study of stroke patients, lower SUA levels on admission were found to be associated with poststroke depression at discharge [17]. In contrast, studies on the relationship between SUA levels and mental health have remained controversial. Li et al. found that adolescent depressive patients have higher SUA levels than controls [18]. There may be a complex relationship between SUA and epilepsy. A recent study reported a U-shaped association between SUA levels and epileptic seizures after cerebral infarction [19]. Some anti-epileptic drugs (AEDs) such as carbamazepine and phenobarbitone have been found to be associated with the levels of SUA in PWE [20].

We hypothesized that lower SUA levels may be related to psychiatric comorbidities in epilepsy. Thus, the aim of this study was to examine the association of SUA levels with depressive and anxiety symptoms among PWE. This study would add new knowledge on correlates of depressive and anxiety symptoms among PWE, apart from previous studies’ findings mainly on sociodemographic and epilepsy-related factors of these psychiatric comorbidities [17, 21].

## Methods

### Study sample

Study subjects were recruited from November 2017 to December 2019 among patients who were treated and followed up at the epilepsy clinic, Department of Neurology, The First Hospital of Jilin University. Epilepsy diagnosis was determined with the 2017 International League Against Epilepsy (ILAE) classification by a neurologist. The following inclusion criteria were used: (1) age ≥ 18 years old; (2) a definite epilepsy diagnosis; (3) without missing data on serum uric acid or psychological scale score; (4) written informed consent was provided. The exclusion criteria comprised: (1) a neurological disease other than epilepsy (e.g., dementia, Parkinson’s disease); (2) medical history of major psychiatric disorders; (3) a serious physical disease (e.g., significant hepatic, renal, or cardiopulmonary condition) or substance abuse history over the past 12 months. Overall, 320 participants were included in the final analysis.

### Data collection

Two trained interviewers used a structured questionnaire to collect demographic and clinical data during a face-to-face interview. We recorded the following data: age, sex, body mass index (BMI), marriage status, education level, occupation, residence, per capita monthly family income, age at seizure onset, epilepsy duration, seizure type, seizure frequency in the last year, treatment type and > 50% nocturnal seizures. Body mass index was calculated as kg/m2. The Neurological Disorders Depression Inventory for Epilepsy [22] (NDDI-E; Chinese version) and Generalized Anxiety Disorder-7 15 [23] (GAD-7; Chinese version) scales were used as screening tools for depressive and anxiety symptoms for PWE. All psychological evaluations were performed by two trained clinicians who were blinded to the data after receiving standardized training. Serum uric acid levels were measured.

### Assessment of depressive symptoms

The NDDI-E scale [22] was used to assess the depressive symptoms of each patient. The NDDI-E scale is a rapid and user-friendly clinical instrument to screen for depressive symptoms in PWE [22]. The C-NDDI-E was validated in Chinese by Dong Zhou et al. and the Cronbach’s α coefficient for the C-NDDI-E was 0.825 [24].. It consists of six self-rated questions, with scores ranging from 1 to 4. The total score is obtained by summing the scores for the 6 questions; a higher C-NDDI-E score indicates more serious depressive symptoms, and a score higher than 12 scale indicates depressive symptoms in PWE [24]. The C-NDDI-E is not a substitute for clinical interview or Diagnostic and Statistical Manual (4th ed.) diagnosis, but it is a reliable, validated, and widely used self-reported measure of depressive symptoms in mainland China [21].

### Assessment of anxiety symptoms

The GAD-7 scale [23] is a valid and efficient tool for screening anxiety disorders and assessing their severity in clinical practice. In this study, the Chinese version of the GAD-7 was used to screen the comorbidity of anxiety symptoms in PWE, and the Cronbach’s α coefficient for the GAD-7 was 0.888 [25]. This questionnaire includes seven self-rating questions, with scores ranging from zero to three. A cutoff of > 6 indicates anxiety symptoms in patients [25].

### Assessment of uric acid

Blood samples were collected from each patient and assessed to detect SUA levels in the Clinical Laboratory of the First Hospital of Jilin University. SUA was measured by using enzymatic colorimetry and a biochemical analyser (7600–210, Hitachi, Japan). Total participants were divided into three groups according to tertiles of SUA levels, which ensured the most categories with adequate numbers of patients per subgroup. The cut-off values for stratification on the SUA level into tertiles were: (T1) *n*=107, ≤ 293 μmol/L; (T2) *n*=107, 294–374 μmol/L; (T3) *n*=106, ≥ 375 μmol/L.

### Statistical analyses

Continuous variables were presented as the means ± standard deviation (SD) or median and interquartile range, according to the normal or nonnormal distribution of the data tested by the Kolmogorov-Smirnov test. Categorical variables were presented as the frequencies and percentages. Student’s t test and analysis of variance (ANOVA) were used for the normally distributed continuous variables, while the Mann-Whitney U-test and Kruskal-Wallis tests were employed for the asymmetrically distributed variables. Chi-square tests were used for categorical variables. Additionally, post-hoc least significant difference (LSD) test and pairwise group comparisons were performed to investigate differences between any two groups using the Bonferroni correction, and *P* < 0.017 was deemed statistically significant in bonferroni correction. The associations of psychiatric comorbidities with SUA levels and other corresponding factors in PWE were assessed by binary logistic regression models and adjusted by related risk factors (*P* < 0.05). We also tested for a linear trend across SUA tertiles in logistic regression models. Odds ratios (ORs) with 95% confidence intervals (CIs) in logistic regression models are presented. Analysis was performed with SPSS for Windows, version 22.0 (SPSS Inc., Chicago, IL, USA). *P* < 0.05 was deemed statistically significant, and all statistical tests were two-sided.

## Results

The sample comprised 320 subjects, 41.9% (*n*=134) of which were female, with a mean (SD) age of 37.88 (15.94) years. The mean SUA level was 340.08 μmol/L. Depressive symptoms were prevalent in 20.3% (*n*=65) of the patients and anxiety symptoms in 25.63% (*n*=82). Demographic and clinical characteristics of the study sample are described in Table 1. All subjects were divided into three subgroups based on tertiles of SUA levels. General characteristics of subjects by the tertiles of SUA are presented in Table 1. A lower SUA tertile was significantly associated with higher C-NDDI-E and GAD-7 scores compared to higher tertiles (*p*=0.001, and *p*= 0.002). Patients in the tertile 1 group had higher C-NDDI-E scores than those in tertile 2 and tertile 3. Patients in the tertile 1 group had higher GAD-7 scores that those in tertile 3. Additionally, there were significant differences in age, gender, BMI, marital status, educational level, age at onset and seizure frequency over the last year between patients with the low, moderate, and high SUA levels. However, residence, disease duration, seizure type, monotherapy and >50% nocturnal seizures were not different between patients with the low, moderate, and high SUA levels.

**Table 1:** Demographic and clinical characteristics of PWE according to SUA levels

Variables	All patients	SUA tertiles			*P*	Tertile 1 vs Tertile 2	Tertile 1 vs Tertile 3	Tertile 2 vs Tertile 3
		Tertile 1, *n*=107 (≤ 293)	Tertile 2, *n*=107 (294–374)	Tertile 3, *n*=106 (≥ 375)				
SUA (umol/L), mean±SD	340.08 ± 93.05	246.16 ± 34.50	330.18 ± 22.96	444.87 ± 66.30	<0.001^**d**^	*p*<0.001^**e**^	<0.001^**e**^	<0.001^**e**^
C-NDDI-E score, median (IQR)	7 (6, 10)	8, (6, 13)	7 (6, 10)	7 (6, 9)	<0.001^**c**^	0.016^**b**^	<0.001^**b**^	0.236^**b**^
GAD-7 score, median (IQR)	3 (1, 7)	5 (1, 9)	3 (0, 6)	2 (0, 4)	0.002^**c**^	0.020^**b**^	<0.001^**b**^	0.302^**b**^
Age (Years), mean±SD	37.88 ± 15.94	40.26 ± 16.35	40.63 ± 16.65	32.71 ± 13.48	<0.001^**d**^	0.864^**e**^	<0.001^**e**^	<0.001^**e**^
Gender-female, n(%)	134 (41.9)	77 (72.0)	40 (37.4)	17 (16.0)	<0.001^**a**^	<0.001^**a**^	<0.001^**a**^	<0.001^**a**^
BMI (Kg/M2), mean±SD	23.48 ± 3.82	22.43 ± 3.57	23.74 ± 3.53	24.28 ± 4.13	0.001^**d**^	0.011^**e**^	<0.001^**e**^	0.293^**e**^
Marital status-married, n(%)	205 (64.1)	78 (72.9)	69 (64.5)	58 (54.7)	0.022^**a**^	0.185^**a**^	0.006^**a**^	0.146^**a**^
Educational level, n(%)					<0.001^**a**^	0.088^**a**^	<0.001^**a**^	0.023^**a**^
University and above	95 (29.7)	25 (23.4)	27 (25.2)	43 (40.6)				
Middle school	193 (60.3)	62 (57.9)	71 (66.4)	60 (56.6)				
Primary school and below	32 (10)	20 (18.7)	9 (8.4)	3 (2.8)				
Residence-rural area, n(%)	115 (35.9)	45 (42.1)	31 (29.0)	39 (36.8)	0.133^**a**^	0.046^**a**^	0.432^**a**^	0.224^**a**^
Per capita monthly family income (Yuan), n(%)					0.098^**a**^	0.745^**a**^	0.057^**a**^	0.050^**a**^
<1000	31 (9.7)	14 (13.1)	12 (11.2)	5 (4.7)				
1000–5000	230 (71.9)	75 (70.1)	80 (74.8)	75 (70.8)				
>5000	59 (18.4)	18 (16.8)	15 (14.0)	26 (24.5)				
Age at onset (Years),median (IQR)	30 (18, 46)	30 (18, 47)	36 (19, 50)	24 (17, 39)	0.005^**c**^	0.406^**b**^	0.019^**b**^	0.002^**b**^
Disease duration (Years),median (IQR)	2 (1, 6)	2 (1, 7)	2 (1, 5)	2 (1, 5)	0.55^**c**^	0.409^**b**^	0.301^**b**^	0.841^**b**^
Seizure type-focal onset, n(%)	266 (83.1)	93 (86.9)	86 (80.4)	87 (82.1)	0.415^**a**^	0.196^**a**^	0.369^**a**^	0.751^**a**^
Seizure frequency over the last year, n(%)					0.015^**a**^	0.151^**a**^	0.002^**a**^	0.279^**a**^
Seizure-feee	63 (19.7)	20 (18.7)	21 (19.6)	22 (20.8)				
1–11	164 (51.2)	44 (41.4)	56 (52.3)	64 (60.4)				
>12	93 (29.1)	43 (40.2)	30 (28.0)	20 (18.9)				
Treatment-monotherapy, n(%)	286 (89.4)	93 (86.9)	96 (89.7)	97 (91.5)	0.548^**a**^	0.523^**a**^	0.280^**a**^	0.654^**a**^
>50% nocturnal seizures, n(%)	120 (37.5)	38 (35.5)	38 (35.5)	44 (41.5)	0.581^**a**^	1^**a**^	0.369^**a**^	0.369^**a**^

*SUA* serum uric acid, *C-NDDI-E* Chinese Version of Neurological Disorders Depression Inventory for Epilepsy, *GAD-7* Generalized Anxiety Disorder-7, *BMI* body mass index, *PWE* patients with epilepsy, *IQR* interquartile range, *SD* standard deviation, *LSD* least significant difference

^a^Chi-square tests, ^b^Mann–Whitney U-test, ^c^Kruskal-Wallis tests, ^d^ANOVA, ^e^Post-hoc LSD tests were used in Table 1. Bonferroni correction was also used, and *P* < 0.017 was deemed statistically significant

The characteristics of patients with and without depressive/anxiety symptoms are summarized in Table 2. Patients with depressive symptoms exhibited significantly lower serum UA levels compared to those without depressive symptoms (*p*< 0.001). Age, gender, educational level and per capita monthly family income showed significant differences between the group of patients with and without depressive symptoms. SUA levels of patients with anxiety symptoms were significantly lower than those of patients without anxiety symptoms (*p*< 0.001). Gender, educational level, and seizure frequency in the last year were significantly different between patients with and without anxiety symptoms.

**Table 2:** Demographic and clinical characteristics of PWE according to depression and anxiety

Variables	Depressive symptoms			Anxiety symptoms		
	With depressive symptoms (*n*=65)	Without depressive symptoms (*n*=255)	*P*	With anxiety symptoms (*n*=82)	Without anxiety symptoms (*n*=238)	*P*
SUA (umol/L), mean±SD	288.52 ± 60.05	353.22 ± 95.46	< 0.001^**a**^	309.39 ± 83.26	350.65 ± 94.06	< 0.001^**a**^
Age (Years), mean±SD	42.77 ± 16.60	36.64 ± 15.56	0.005^**a**^	36.77 ± 16.04	38.26 ± 15.92	0.464^**a**^
Gender-female, n (%)	38 (58.5)	96 (37.6)	0.002^**c**^	47 (57.3)	87 (36.6)	0.001^**c**^
BMI (Kg/M2), mean±SD	23.21 ± 3.29	23.55 ± 3.95	0.516^**a**^	22.78 ± 3.58	23.73 ± 3.88	0.053^**a**^
Marital status-married, n (%)	46 (70.8)	159 (62.4)	0.207^**c**^	50 (61.0)	155 (65.1)	0.499^**c**^
Educational level, n (%)			< 0.001^**c**^			0.005^**c**^
University and above	10 (15.4)	85 (33.3)		15 (18.3)	80 (33.6)	
Middle school	40 (61.5)	153 (60.0)		53 (64.6)	140 (58.8)	
Primary school and below	15 (23.1)	17 (6.7)		14 (17.1)	18 (7.6)	
Residence-rural area, n (%)	24 (36.9)	91 (35.7)	0.853^**c**^	33 (40.2)	82 (34.5)	0.346^**c**^
Per capita monthly family income (Yuan), n (%)			0.026^**c**^			0.135^**c**^
<1000	12 (18.5)	19 (7.5)		11 (13.4)	20 (8.4)	
1000–5000	43 (66.2)	187 (73.3)		61 (74.4)	169 (71.0)	
>5000	10 (15.4)	49 (19.2)		10 (12.2)	49 (20.6)	
Age at onset (Years),median (IQR)	37 (19, 48)	29 (16, 46)	0.085^**b**^	29 (18, 45)	30 (18, 47)	0.552^**b**^
Disease duration (Years),median (IQR)	2 (1, 7)	2 (1, 6)	0.786^**b**^	2 (1, 5)	2 (1,6)	0.557^**b**^
Seizure type-focal onset, n (%)	54 (83.1)	212 (83.1)	0.991^**c**^	68 (82.9)	198 (83.2)	0.956^**c**^
Seizure frequency over the last year, n (%)			0.44^**c**^			0.03^**c**^
Seizure-feee	11 (16.9)	52 (20.4)		10 (12.2)	53 (22.3)	
1–11	31 (47.7)	133 (52.2)		40 (48.8)	124 (52.1)	
>12	23 (35.4)	70 (27.5)		32 (39.0)	61 (25.6)	
Treatment-monotherapy, n (%)	57 (87.7)	229 (89.8)	0.622^**c**^	73 (89.0)	213 (89.5)	0.905^**c**^
>50% nocturnal seizures, n (%)	27 (41.5)	93 (36.5)	0.451^**c**^	34 (41.5)	86 (36.1)	0.39^**c**^

*SUA* serum uric acid, *BMI* body mass index, *PWE* patients with epilepsy, *IQR* interquartile range, *SD* standard deviation

Student’s t tests^a^, Mann–Whitney U-tests^b^ and Chi-square tests^c^ were used in Table 2

The association between SUA levels and depressive symptoms are presented in Table 3. Lower SUA levels were found to increase the risk of depressive symptoms among PWE, with the highest tertile group as the reference group. The first SUA tertile had a strong association with depressive symptoms in an unadjusted logistic regression model (OR 8.102, 95% CI 3.237–20.279, *p* < 0.001). The second SUA tertile was also associated with depressive symptoms (OR 4.819, 95% CI 1.881–12.349, *p* = 0.001). After adjusting for cofounding factors in model 2^b^ and model 3^c^, the association mentioned above still remained. The first tertile of SUA remained independently associated with the risk of depressive symptoms, with the third tertile group as the reference group after adjusting for confounders (model 2^b^: OR = 4.676, 95% CI = 1.650~13.247, *P* = 0.004; model 3^c^: OR = 4.694, 95% CI = 1.643~13.413, P = 0.004). Similarly, the second SUA tertile remained independently associated with the risk of depressive symptoms (model 2^b^: OR = 3.412, 95% CI = 1.272~9.148, *P* = 0.015; model 3^c^: OR = 3.440, 95% CI = 1.278~9.256, *P* = 0.014). A test for linear trend across SUA tertiles was statistically significant (*p*=0.005) in the fully adjusted logistic regression model 3c.

**Table 3:** Binary logistic regression analyses for the association between SUA levels and depression in PWE

Tertiles of SUA levels (umol/L)	Model 1a		Model 2b		Model 3c	
	OR (95% CI)	P	OR (95% CI)	P	OR (95% CI)	P
Tertile 1, *n*=107 (≤ 293)	8.102 (3.237, 20.279)	< 0.001	4.676 (1.650, 13.247)	0.004	4.694 (1.643, 13.413)	0.004
Tertile 2, *n*=107 (294–374)	4.819 (1.881, 12.349)	0.001	3.412 (1.272, 9.148)	0.015	3.440 (1.278, 9.256)	0.014
Tertile 3, *n*=106 (≥ 375)	1.00 (Reference)	–	1.00 (Reference)	–	1.00 (Reference)	–
P for trend	<0.001		0.005		0.005	

*OR* odds ratio, *CI* confidence interval, *SUA* serum uric acid, *PWE* patients with epilepsy

Binary logistic regression analyses were used in Table 3

^b^ adjusted for age, gender, BMI, marital status, educational level, and Per capita monthly family income

^C^ adjusted for the terms in model2^b^ plus age at onset and Seizure frequency over the last year

The association between SUA levels and anxiety symptoms are described in Table 4. In the unadjusted logistic regression model, the first SUA tertile was significantly associated with the risk of anxiety symptoms compared with the third tertile (OR = 2.692, 95% CI = 1.415~5.123, *P* = 0.003). However, the association mentioned was no longer significant after adjusting for confounders (model 2^b^: OR = 1.616, 95% CI = 0.736~3.548, *P* = 0.231; model 3^c^: OR = 1.556, 95% CI = 0.699~3.464, *P* = 0.279). The second SUA tertile was not associated with the risk of anxiety symptoms compared with the third tertile in the unadjusted and adjusted logistic regression models (model 1^a^: OR = 1.569, 95% CI = 0.801~3.074, *P* = 0.189; model 2^b^: OR = 1.294, 95% CI = 0.628~2.666, *P* = 0.486; model 3^c^: OR = 1.265, 95% CI = 0.607~2.635, *P* = 0.53). A test for linear trend across SUA tertiles was not significant (*p*=0.279) in the fully adjusted logistic regression model 3c.

**Table 4:** Binary logistic regression analyses for the association between SUA levels and anxiety in PWE

Tertiles of SUA levels (umol/L)	Model 1a		Model 2b		Model 3c	
	OR (95% CI)	P	OR (95% CI)	P	OR (95% CI)	P
Tertile 1, *n*=107 (≤ 293)	2.692 (1.415, 5.123)	0.003	1.616 (0.736, 3.548)	0.231	1.556 (0.699, 3.464)	0.279
Tertile 2, *n*=107 (294–374)	1.569 (0.801, 3.074)	0.189	1.294 (0.628, 2.666)	0.486	1.265 (0.607, 2.635)	0.53
Tertile 3, *n*=106 (≥ 375)	1.00 (Reference)	–	1.00 (Reference)	–	1.00 (Reference)	–
P for trend	0.002		0.231		0.279	

*OR* odds ratio, *CI* confidence interval, *SUA* serum uric acid, *PWE* patients with epilepsy

Binary logistic regression analyses were used in Table 4

^b^ adjusted for age, gender, BMI, marital status, and educational level

^C^ adjusted for the terms in model2^b^ plus age at onset and Seizure frequency over the last year

## Discussion

To our knowledge, this is the first study to investigate the relationship between SUA levels and mental health among PWE. In our cohort of PWE, lower SUA levels were significantly associated with a higher risk of depressive symptoms but not with a higher risk of anxiety symptoms in epilepsy.

A complex relationship has been reported between SUA and epilepsy. SUA levels greater than 322 μmol/L or less than 248 μmol/L have been associated with an increased risk of developing epileptic seizures after cerebral infarction compared to SUA levels of 248–322 μmol/L, indicating a U-shaped dose-effect relationship between the SUA level and epilepsy after cerebral infarction [19]. A previous study reported that treatment with carbamazepine and phenytoin was related with low SUA levels, and phenobarbitone and sodium valproate were associated with elevated SUA [20]. Allopurinol has been proposed as a safe and effective adjuvant agent in refractory epilepsy that could decrease the frequency of seizures by influencing purine metabolism pathways and SUA levels [26]. We found in this study that epilepsy-related factors including age at seizure onset and seizure frequency over the last year were associated with SUA levels in PWE. Similarly, age at seizure onset and seizure frequency have been reported as risk factors for depression in PWE [4, 27]. These results may explain the link between SUA levels and depressive symptoms in PWE. Considering these findings, epileptic seizure and SUA affect each other, and the interaction is responsible for the development of epilepsy [19]. Given that lowered SUA levels have also been observed in depression and anxiety disorders, there may be a complex relationship between SUA, mental illness and epilepsy. Depression and anxiety are highly prevalent psychiatric comorbidities in PWE. Thus, we aimed to investigate the association between SUA, depressive and anxiety symptoms in epilepsy.

The present study revealed that the prevalence of depressive symptoms was higher among PWE with lower SUA levels. We are unaware of any similar studies showing an association of SUA levels with depressive and anxiety symptoms in PWE. Our findings about the benefits of high SUA levels on depressive symptoms are consistent with several previous studies [15–17, 28]. A previous study demonstrated that SUA levels were significantly lower in individuals with depression or anxiety compared with healthy controls, and these data suggested that lowered SUA levels are a characteristic of depression [15]. A national population-based survey among 10,524 participants aged 45 years and above demonstrated that higher SUA levels were correlated with a decreased risk of depression in men but not in women [28]. In a Chinese cohort of stroke survivors, lower SUA levels on admission have been associated with an increased risk of poststroke depression at discharge [17]. However, studies on the association of SUA levels with depression remain controversial [18, 29]. Gao et al. reported that higher SUA levels on admission were related with the occurrence of major poststroke depression within 3 months of stroke [29]. Li et al. found higher SUA levels in adolescent depressive patients, which could be used as a biomarker for the early diagnosis of depression in adolescents [18]. Two recent meta-analyses reported lower SUA levels in depression with high heterogeneity (I^2^±90%), indicating that the results have been inconsistent [30, 31].

The neuroprotective effects of SUA have been identified due to its antioxidant properties. Depression and anxiety disorders may share similar neurobiological mechanisms. However, we found that lower levels of SUA were independently associated with an increased risk of depressive symptoms but not anxiety symptoms after adjusting for related risk factors, which was not in line with a recent study [16]. Possible explanations may be the relatively small sample size in our study and the different diagnostic criteria of anxiety disorders among studies. Further well-designed prospective cohort studies with larger sample sizes are required to confirm the association between SUA levels and the risk of anxiety symptoms in patients with epilepsy.

Evidence indicated that elevations in SUA levels may be associated with anticipation of challenging stress situations [32]. A previous study found that SUA levels rose significantly in men who lose their jobs, while successful re-employment resulted in a return to normal SUA levels [33]. Additionally, stress situation is a common reported seizures precipitants in PWE, and Moon et al. found that the strongest predictor for perceived stress was depressive symptom in PWE [34]. These data supported the association between SUA levels, stress situation and depressive symptoms.

The present study illustrated that the risk of depressive symptoms was higher among participants with lower SUA levels. However, SUA levels were not associated with the risk of anxiety symptoms among PWE. We could not determine the potential mechanisms for the lower SUA levels in depressive symptoms among PWE. The current findings supporting a protective role of high SUA levels against depressive symptoms may be explained partly by the protective roles played by SUA in oxidative damage [35–37]. Oxidative damage and oxidative stress have been reported to play key roles in the development of depression and epilepsy [38–40]. Uric acid (UA) is known as an important natural antioxidant in the human body and plays a key role in the antioxidant capacity against oxidative and radical injury [12]. Lower SUA levels indicate lower antioxidant defences. Evidence has shown that lowered SUA levels lead to increased susceptibility to oxidative damage in the vulnerable brain due to its high oxygen consumption [41]. The brain is one of the most vulnerable organs due to its high metabolic rate. Reactive oxygen species (ROS) may lead to DNA and protein damage in the brain by reacting with these macromolecules. This damage may play an active role in the pathophysiology of affective disorders and make individuals susceptible to developing depression [42, 43]. Additionally, UA is a marker and the final product of purine metabolism. The purinergic system has been reported to be involved in the pathophysiology of affective disorders and is considered to influence mental health, cognition and sleep by the neurotransmitter adenosine triphosphate (ATP) and the neuromodulator adenosine, both of which are upstream metabolites of uric acid [44, 45].

Several limitations should be noted in our study. First, definitive conclusions about causality cannot be made given that our study was cross-sectional, and further prospective investigation is required to confirm our findings. Second, depressive and anxiety symptoms were diagnosed according to the scores of the C-NDDI-E and GAD-7 scales in this study. Clinical diagnosis is clearly the gold standard. While the C-NDDI-E is not a substitute for clinical interview or Diagnostic and Statistical Manual (4th ed.) diagnosis, it is a reliable, validated, and widely used self-reported measure of depression in mainland China [21, 24]. The Chinese version of the GAD-7 has also been validated in Chinese [25]. Third, the possibility of residual confounding factors cannot be excluded, although we adjusted for factors that could influence SUA levels or depressive symptoms among PWE. An important related risk factor is the anticonvulsants that may influence uric acid levels, anxiety and depressive symptoms in patients. Different types of anticonvulsants may have various impacts on uric acid levels, anxiety and depressive symptoms. Additionally, we divided all individuals into three groups according to tertiles of SUA levels, which was convenient for the statistical analysis. However, it may not have been a statistically or absolutely correct practice. The lack of a healthy control group was another limitation.

## Conclusion

In conclusion, we found that lower SUA levels were independently associated with depressive symptoms but not with anxiety symptoms among PWE. These findings could offer in vivo evidence that SUA may play a role in the aetiology of depression. Our study also suggested that lower SUA levels might be used as an indicator of depressive symptoms among PWE. Further well-designed prospective cohort studies are required to determine the causality of the associations and to further clarify the mechanisms of SUA in depression.

## Data Availability

The deidentified database used in the current study is available from the corresponding authors on reasonable request.

## Abbreviations

SUA: Serum uric acid
PWE: Patients with epilepsy
C-NDDI-E: Chinese Version of Neurological Disorders Depression Inventory for Epilepsy
GAD-7: Generalized Anxiety Disorder-7
ILAE: International League Against Epilepsy
ORs: Odds ratios
CIs: Confidence intervals
ROS: Reactive oxygen species
ATP: Neurotransmitter adenosine triphosphate
